# Calibration of a Dust Scattering Instrument Using Tomographic Techniques and Its Application to a Dust Sensor Instrument

**DOI:** 10.3390/s23115036

**Published:** 2023-05-24

**Authors:** David Santalices, Mateo Martínez-García, Jesús Belmar, Daniel Benito, Susana Briz, Juan Meléndez, Antonio J. de Castro

**Affiliations:** 1 LIR—Infrared Laboratory, Department of Physics, Universidad Carlos III de Madrid, 28911 Leganés, Spain; 2 Science Faculty, Universidad Nacional de Educación a Distancia (UNED), 28040 Madrid, Spain

**Keywords:** scattering sensor, nephelometer, angular weighting function, scattering of particles, Martian dust, radon transform, tomography

## Abstract

The characterization of suspended dust near the Martian surface is extremely relevant to understand the climate of Mars. In this frame, a Dust Sensor instrument, an infrared device designed to obtain the effective parameters of Martian dust using the scattering properties of the dust particles, was developed. The purpose of this article is to present a novel methodology to calculate, from experimental data, an instrumental function of the Dust Sensor that allows solving the direct problem and providing the signal that this instrument would provide given a distribution of particles. The experimental method is based on recording the signal measured when a Lambertian reflector is gradually introduced into the interaction volume at different distances from the detector and source and applying tomography techniques (inverse Radon transform) to obtain the image of a section of the interaction volume. This method provides a complete mapping of the interaction volume experimentally, which determines the Wf function. The method was applied to solve a specific case study. Among the advantages of this method, it should be noted that it avoids assumptions and idealizations of the dimensions of the volume of interaction and reduces the time required to carry out simulations.

## 1. Introduction

Dust suspended in the atmosphere of Mars is the main factor that governs its meteorology and climate; therefore, the characterization of particles in suspension, in particular their density and effective parameters (modal radius and variance), is essential to understand and predict not only the Martian climate [1] but to evaluate its influence in Entry, Descent and Landing (EDL) systems [2] and the potential hazards for equipment and human crews [3].

Over the past 50 years, many instruments (both orbiters and in situ instruments [4]) have measured the opacity of the Martian atmosphere in different spectral ranges and conditions, resulting in multiple studies of dust size parameters [5,6,7]. However, it is known that there are discrepancies between the particle size distributions and the dust densities of different models; therefore, there is still a need for research in this field [8].

In this context, the use of in situ instruments capable of characterizing suspended dust near the Martian surface is extremely relevant to understand the climate of Mars and its atmospheric dynamics, as well as to provide ground truth for the models and in-orbit instruments [9].

**Figure 1:**
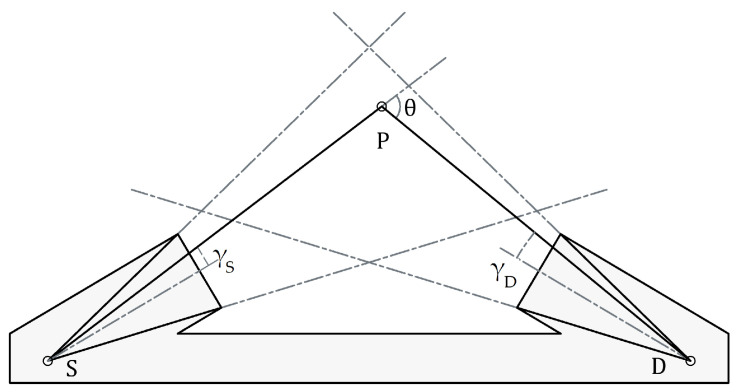
A diagram of the Dust Sensor. S is the IR source, D the forward detector and P is a generic scattering point, located at angles $\gamma_{S}$ and $\gamma_{D}$ with, respectively, the axes of the source and the detector.

Such an instrument is the Dust Sensor [10], a device designed to obtain the effective parameters of Martian dust. Its operating principle is based on the fact that the scattering of light by dust particles depends not only on the effective parameters of the particles but also on the wavelength of the incident light and its scattering angle. Therefore, the parameters can be determined by measuring the light scattered by the particles at different wavelengths and different scattering angles and solving the inverse problem. For that reason, the Dust Sensor instrument consists of a broadband IR emitter and two detectors of PbSe and PbS. Each pair of PbSe–PBS detectors is placed in different orientations to measure the light scattered by the particles in two different directions (forward and backward) and at two wavelengths (3–5 μm and 1–3 μm, respectively); the effective parameters of suspended dust can be determined by solving an inverse problem. Figure 1 shows a diagram of the Dust Sensor, where *S* is the IR source, *D* is one of the detectors (forward) and *P* is the point that scatters the light with a scattering angle of θ.

Obtaining the actual dust distribution parameters from the instrument signal requires first solving the direct problem; that is, given a dust particle distribution, determining what signal the instrument would provide. Since the signal not only depends on the particle distribution but also on the geometry and spectral response of the instrument, it is essential to calculate the contribution of the instrument’s characteristics to the signal. The objective of this article is to obtain an instrumental function that describes the instrument response in order to obtain a method that calculates, in a straightforward way, the signal that the instrument would provide for a given a distribution of particles.

What is generally done is to define an ideal sampling volume as the intersection between the cone corresponding to the light emitted by the source and the Field of View (FoV) cone of the detector. This ideal volume is then used to define the direct model, either by Monte Carlo methods [11] or by angular weighting functions that encompass the behavior of the instrument as a function of the scattering angle [12,13,14]. These methods require further experimental calibration, either by media with known particle distributions [12] or by targets of known emissivity [15].

However, the emission pattern of the source and the angular response of the detector are usually nonideal, and the sampling volume resulting from both will often be irregular in shape; in addition, the response of the instrument will not be uniform within that volume. To solve the nonidealities in integrating nephelemoters, some studies include a truncated angular weighting function [16,17,18]. In a previous work [19], the authors proposed a method to solve the problem of the idealization of the volume of interaction by defining an angular weight function, $W_{f}\left(\theta \right)$, based on the choice of an appropriate coordinate system for the instrument, which depends only on the scattering angle θ and groups all the geometric factors of the instrument, without assuming ideal emission or FoV patterns.

In this work, a novel experimental method is proposed to obtain $W_{f}$ and to calibrate this type of instrument. The method is based on recording the signal measured when a Lambertian reflector is gradually introduced into the interaction volume at different distances from the detector and source.

At each distance, the instrument is rotated, and by applying tomography techniques (inverse Radon transform), we are able to obtain a section of the interaction volume from the scalar outputs provided by the detector. Repeating the process for different distances, a complete mapping of the interaction volume is obtained. This determines experimentally the $W_{f}$ function. Knowledge of this function and the emission pattern of the reflector makes it possible to calculate the irradiance that reaches the detector and thus calibrate the instrument by relating its output (Volts) to the radiative input in the detector (Watts).

Although other authors [15] have also experimentally obtained the instrument response by measuring a reflector at different distances from the instrument, they need to assign an effective scattering angle to each of these distances. The main novelty of the method we propose is the use of tomographic techniques to obtain sections of the interaction volume from the signal provided by a point detector. Therefore, our method avoids the approach that involves defining an effective scattering angle. Furthermore, by using $W_{f}$, it does not have the problems associated with the idealization of the emission pattern of the IR source and the FoV of the detector.

The structure of this paper is as follows. Section 2 explains the theoretical foundations of this work. The proposed methodology to calculate $W_{f}$ from experimental data and the set up required for the measurements are developed in Section 3. The partial results of applying the methodology to the Dust Sensor as well as the final $W_{f}$ function are presented in Section 4. In addition, Section 4 includes an application that shows the usefulness of this methodology to solve a direct problem: to calculate the signal that the Dust Sensor would provide for a distribution of spherical particles. Finally, Section 5 is devoted to discussing the results and summarizing the main conclusions.

## 2. Theoretical Background

A backward/forward scatter instrument consists of a point source and a detector, both usually collimated. A schematics of a forward arrangement can be found in Figure 1.

To calculate the radiant flux reaching the detector, $\Phi_{D}$ [*W*], we can use the volume scattering function, β [m${}^{-1}$sr${}^{-1}$], which depends on the medium (density and type of scattering particles). This function is defined from the radiant intensity dI [W/sr] scattered by a differential volume element dV, when an unpolarized plane wave with an irradiance *E* [W/m^2^] illuminates it:

(1) dI=E(λ)·β(θ,λ)·dV

The spectral radiant flux $d\Phi_{D}\left(\theta ,\lambda \right)$ [W/μm] that reaches the detector from a *dV* at a point P can be expressed as

(2) $$d\Phi_{D}\left(\theta ,\lambda \right)=E\left(\lambda \right)\cdot \frac{\sigma (\gamma_{D})\cdot A}{r^{2}}\cdot \beta \left(\theta ,\lambda \right)\cdot dV$$

where $\frac{A}{r^{2}}$ [sr] is the solid angle defined by the area *A* of the detector as seen from the scattering point *P* at a distance *r*, and $\sigma (\gamma_{D})$ takes into account the angular sensitivity of the detector (for an ideal detector $\sigma \left(\gamma_{D}\right)=\cos\left(\gamma_{D}\right)$). The signal measured by the detector is, therefore,

(3) $$\begin{array}{c} \langle Signal\rangle =\int_{\lambda }d\lambda \int_{V}dV\cdot E\left(x,y,z,\lambda \right)\cdot \frac{\sigma (\gamma_{D}\left(x,y,z\right))\cdot A}{r{\left(x,y,z\right)}^{2}}\cdot \beta \left(\theta ,\lambda \right)g\left(\lambda \right) \end{array}$$

where *g(λ)* is the spectral responsivity [V/W] of the detector. If the irradiance on *P* is written as

(4) $$E\left(x,y,x,\lambda \right)=E_{\lambda }\left(\lambda \right)\cdot E_{0}\left(x,y,z\right)$$

then

(5) $$\begin{array}{c} \langle Signal\rangle =\int_{\lambda }d\lambda \int_{V}dV\cdot VWF\left(x,y,z\right)\cdot \beta \left(\theta ,\lambda \right)E_{\lambda }\left(\lambda \right)g\left(\lambda \right) \end{array}$$

where

(6) $$\begin{array}{c} VWF\left(x,y,z\right)\equiv E_{0}\left(x,y,z\right)\frac{\sigma (\gamma_{D}\left(x,y,z\right))\cdot A}{r{\left(x,y,z\right)}^{2}} \end{array}$$

is a function that groups the terms that depend only on the geometry of the instrument and will be called from now on as volume weighting function (VWF). As explained in detail in a previous work [19], there is a specific coordinate system $\left(q_{1},q_{2},\theta \right)$ that allow us to express the VWF as a one-dimensional function, called angular weighting function ($W_{f}$), that only depends on the scattering angle θ. This $W_{f}\left(\theta \right)$ function can be calculated through integration on the other two coordinates, $q_{1}$ and $q_{2}$:

(7) $$W_{f}\left(\theta \right)=\int dq_{1}\int dq_{2}\;VWF\left(q_{1},q_{2},\theta \right)\cdot J\left(q_{1},q_{2},\theta \right)$$

where $J(q_{1},q_{2},\theta )$ is the Jacobian determinant.

Using $W_{f}\left(\theta \right)$, it is possible to express the integral in Equation (5) as a one-dimensional integral of a function that only depends on the instrument,

(8) $$\langle Signal\rangle =\int_{\lambda }d\lambda \int_{\theta }d\theta \cdot W_{f}\left(\theta \right)\cdot \beta \left(\theta ,\lambda \right)E_{\lambda }\left(\lambda \right)g\left(\lambda \right)$$

## 3. Methodology

As mentioned in the introduction, the objective of this work is to experimentally determine the function $W_{f}$ that describes the geometric characteristics of the instrument. This is explained in the following section. Here, the spectral dependence is omitted for simplicity, and it is treated in Section 3.2.

### 3.1. Angular Weighting Function

**Figure 2:**
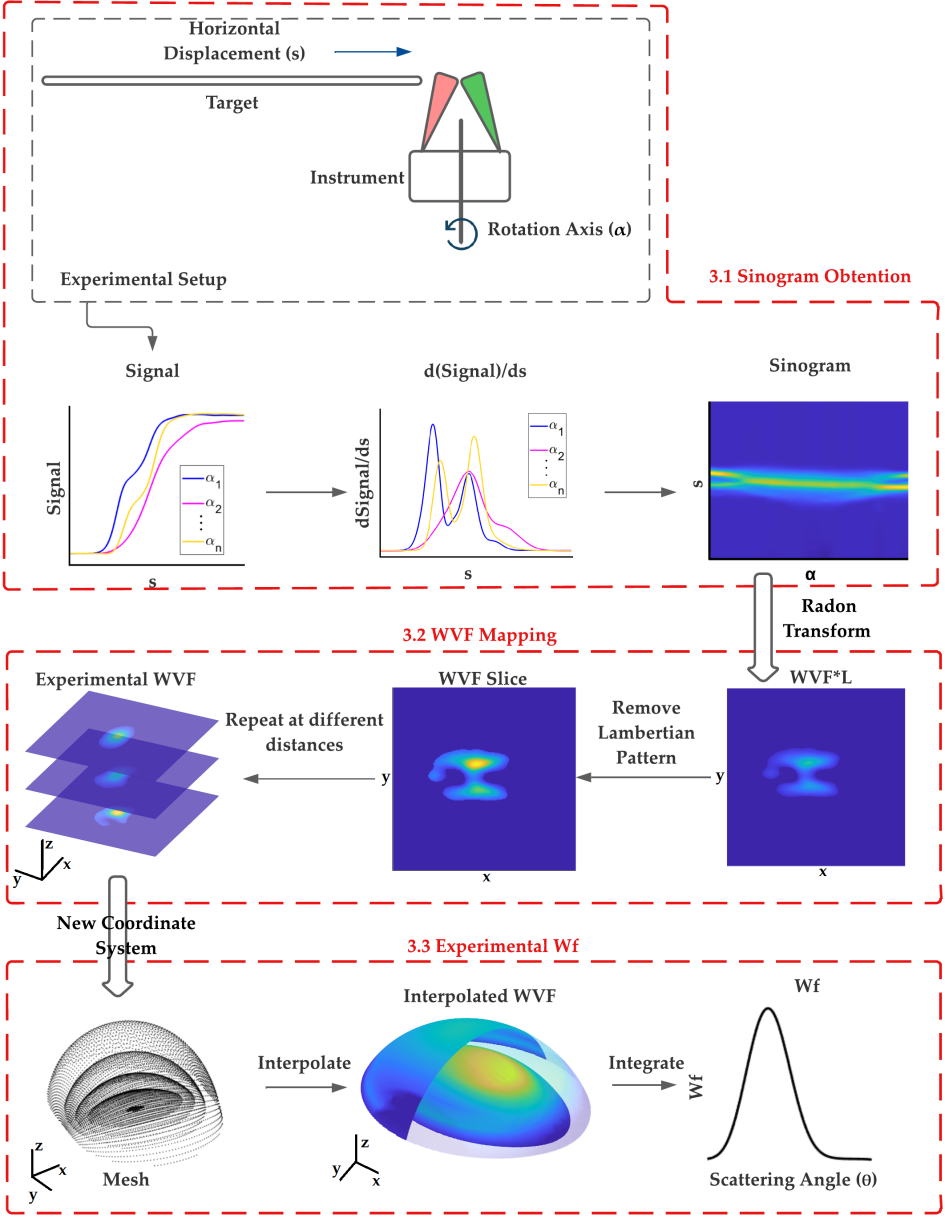
Flowchart that summarize the methodology followed to obtain an experimental $W_{f}$.

**Figure 3:**
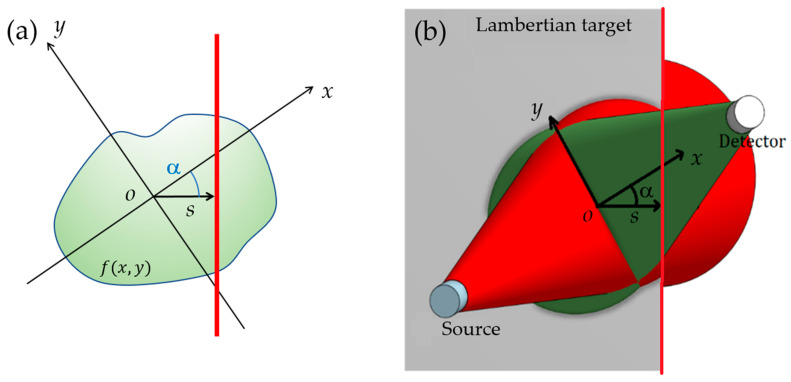
(**a**) Radon transform coordinates. (**b**) A scheme showing the coordinates in our problem (the instrument is seen from the back of the instrument, in the direction of the *z*-axis).

Since $W_{f}$ is calculated by integration of the VWF function (Equation (7)), it is necessary to develop a methodology to map the VWF of the instrument. This has been performed by performing a tomography of the VWF using a Lambertian target. Figure 2 represents a schematic of the procedure followed to apply this methodology. The basic idea is to gradually introduce the target (a flat plate) into the interaction volume of the instrument at a certain distance from it. As the target is introduced into the interaction volume with an increasing horizontal displacement *s*, the signal that reaches the detector increases, since there is more reflective surface within its FoV. The derivative of the signal with respect to *s* provides the contribution to the signal from just the edge of the target (red line in Figure 3). This value corresponds to the line integral of the VWF for a given horizontal displacement *s*. By repeating this procedure for different rotation angles and representing the derivative of the signal for each displacement *s* and angle α, we obtain what is known as the sinogram. To obtain the VWF at that specific distance, it is necessary to apply the inverse Radon transform to the sinogram and eliminate a reflection factor *L* due to the target.

By repeating this procedure at different distances *z*, multiple sections of the VWF are obtained to map this function over the whole of the interaction volume. Finally, interpolating the measured VWF into the coordinate system dependent on θ and integrating over the non-θ coordinates provides the $W_{f}$.

The explanation of the different blocks of the process, depicted in the flowchart in Figure 2, is provided in the following subsections. To begin with, in Section 3.1.1, we describe the experimental setup necessary to carry out the measurements. Then, we detail the blocks that make up the methodology. In Section 3.1.2, we explain how the Radon transform is related to the VWF, as well as the process of obtaining a sinogram; in Section 3.1.3, the VWF mapping procedure is described; and finally, in Section 3.1.4, the method to obtain $W_{f}$ once VWF has been mapped is expounded.

#### 3.1.1. Experimental Setup

The Dust Sensor instrument has been described previously in [10]. It uses a thermoresistive membrane capable of radiating in a broad IR spectral range as the source and has two detectors (PbSe and PbS) for each configuration (forward and backward). In this work, $W_{f}$ was obtained for the PbS detector in the forward configuration. The PbS detector operates in the 1–3 μm spectral band and is enclosed within a parabolic mirror, which reduces its field of view.

A Lambertian target of known reflectivity (Spectralon SPR-99 [20]) and a size of 25 × 25 cm^2^ was used. During the measurement, this target slides along a motorized linear guide in the direction of the *s*-axis. In addition, the Dust Sensor is mounted on a rotating platform that allows measurements to be taken at different angles α. This mechanical setup makes it possible to acquire a sinogram with a spacing of 1 mm on the *s*-axis and a spacing of 8.1${}^{\circ }$ on the α axis. This process is repeated for 11 different distances, ranging from 1 to 10 cm.

To avoid interference from external light sources, the thermoresistive membrane source is modulated and paired with a lock-in amplifier, as detailed in [21]. This causes a change in the spectral signature of the source, which is taken into account in [22].

When the Lambertian target is positioned close to the instrument, internal reflections can occur at the target–instrument interface and potentially reach the detector. To minimize this effect, the surface of the instrument was coated with a high-emissivity (low-reflectance) paint. The edge of the Lambertian target was covered with a high-emissivity tape (ϵ=0.95), thus reducing potential reflections with the target’s edge.

Further information about the spectral characteristics of the emitter and the detector can be found in the Appendix B.

#### 3.1.2. Obtaining the Sinogram

The Radon transform (RT) is an integral transform that relates a function defined on a plane, f(x,y), to its transformed function, R[f], defined in the space of straight lines on the plane. If a line is defined by its minimum distance to the origin, *s*, and its angle with the *x*-axis, α, its equation is xcosα+ysinα−s=0. Figure 3a presents a schematic of both coordinate systems, with the edge of the Lambertian target depicted in red. In Figure 3b both coordinate systems are represented on a diagram of the instrument. In this representation, the instrument (source and detector) is shown in the foreground. The *x*-axis is on the line that joins both elements of the instrument. The angle of rotation of the DS with respect to the horizontal direction is α. The Lambertian target is located in this perspective in a medium plane. The target is introduced into the interaction volume, which is represented by the intersection between the cone corresponding to the FoV of the detector (in green) and that of the emission pattern of the source (in red). The edge of the target is highlighted in red, as in the Figure 3a. A top view of this schematic can be seen in the first section of Figure 2.

The Radon transform assigns to (s,α) the value of the integral of *f* along that line, i.e.,

(9) $$R\left[f\right]\left(s,\alpha \right)=\int_{-\infty }^{+\infty }dx\int_{-\infty }^{+\infty }dy\;f\left(x,y\right)\cdot \delta \left(x\cos\alpha +y\sin\alpha -s\right)$$

It is common to represent the values of R[f](s,α) in a graph with axes α and *s*, called a sinogram.

In our approach, R[f](s,α) is obtained experimentally, as explained next, and then f(x,y) is retrieved by means of the inverse Radon transform.

**Figure 4:**
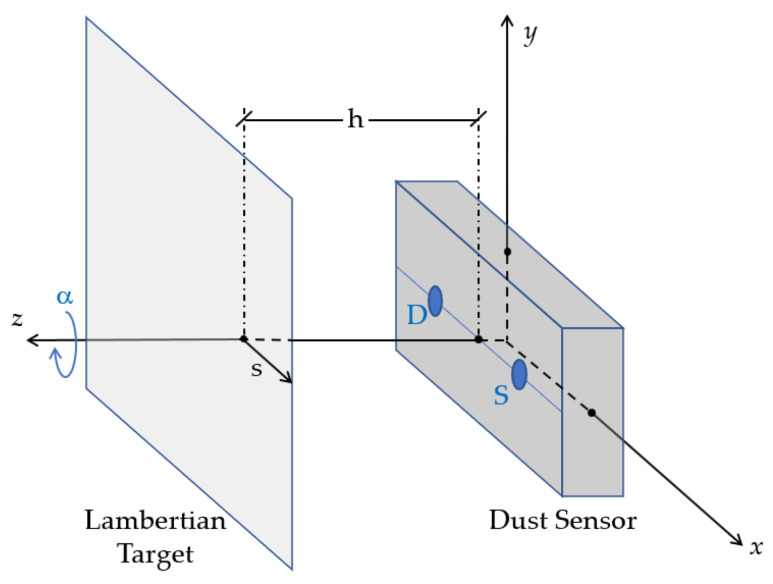
Scheme showing the source, the detector and the Lambertian target, as well as the relationship between the Cartesian and the Radon space coordinates. To acquire the sinogram, the Lambertian target moves in the direction of s, and the instrument rotates around the *z*-axis.

To describe our experimental configuration for R[f](s,α) measurement, we start by defining a coordinate system. The XY plane contains both the source and the detector, with the $\bar{SD}$ line along the *x*-axis, and it is parallel to the Lambertian target, placed at the plane *z* = *h*. The *z*-axis, in turn, coincides with the axis of rotation of the instrument (see Figure 4).

For each distance h, the Lambertian target is introduced along the *s*-axis, measuring continuously until the signal remains constant, which means that the target covers the entire interaction volume (see block 3.1.1. in Figure 4). This process is repeated for an interval of angles α from 0 to 180 degrees.

It is important to note that when the target does not completely cover the interaction volume, the radiant flux received by the detector only comes from the area of the target inserted into the volume. This situation can be described using the Heaviside function H(t). Thus, the radiant flux received by the detector that comes from the area of the Lambertian target within the interaction volume can be expressed as

(10) $$\Phi_{D}\left(s,\alpha \right)=\int_{-\infty }^{+\infty }dx\int_{-\infty }^{+\infty }dy\;VWF\left(x,y,z=h\right)\cdot L\left(x,y,z=h\right)\cdot \left(1-H\left(x\cos\alpha +y\sin\alpha -s\right)\right)$$

where VWF(x,y,z=h) is a section of the VWF contained in the plane z=h, and L(x,y,z=h) is a function that describes the diffuse reflection of the Lambertian target and plays a role analogous to that of β(θ(x,y,z)) in Equation (3). The factor 1−H(xcosα+ysinα−s) is used to restrict the integral to the area of the target embedded in the interaction volume (gray area in Figure 3b), whose boundary is the line xcosα+ysinα−s (red line in Figure 3b).

As the Lambertian targets moves along the *s*-axis, varying the values of *s*, the successive increments in the value of $\Phi_{D}$ are due not only to the edge of the target but to the rest of the area that is gradually introduced into the volume of interaction. The derivative with respect to *s* of Equation (10) provides the contribution of just the edge of the target and therefore the value of the Radon transform of VWF·L. Mathematically, this derivative gives rise to an equation analogous to Equation (9):

(11) $$\frac{d\Phi_{D}}{ds}\left(s,\alpha \right)=\int_{-\infty }^{+\infty }dx\int_{-\infty }^{+\infty }dy\;VWF\left(x,y,z=h\right)\cdot L\left(x,y,z=h\right)\cdot \delta \left(x\cos\alpha +y\sin\alpha -s\right)$$

Therefore, $\frac{d\Phi }{ds}\left(s,\alpha \right)=R\left[f\right]\left(s,\alpha \right)$, being f(x,y)=VWF(x,y,z=h)·L(x,y,z=h).

To summarize, since the signal provided by the instrument is proportional to $\Phi_{D}$, by taking several continuous measurements of the Lambertian target as it enters the interaction volume, varying *s*, (with constant *h* and for a interval of α values from 0° to 180°) a sinogram can be obtained experimentally that is the Radon transform of VWF·L. Repeating this process at different distances, *h*, and applying the inverse Radon transform to each sinogram allows us to achieve a complete mapping of VWF·L.

#### 3.1.3. VWF Mapping

In order to obtain VWF, however, the contribution of the reflector, summarized in the *L* factor, must be removed. In order to do that, it is necessary to find an equation for the radiant flux that reaches the detector. This equation will be the analogue to Equation (2), which was valid for radiation scattered by particles in a volume dV, but now due to diffuse (lambertian) reflection on a dA from the rectangular plate.

**Figure 5:**
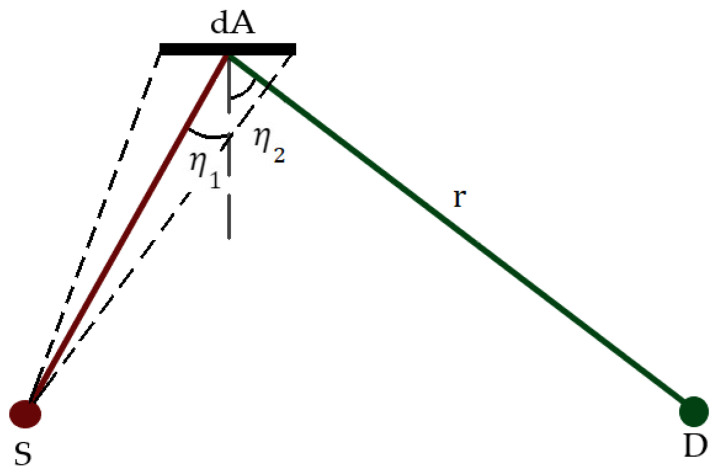
Schematics of the angles involved in the diffuse reflection of the Lambertian source.

If the angles of incidence and reflection at dA are, respectively, $\eta_{1}$ and $\eta_{2}$ (Figure 5), the radiant flux that reaches dA can be written as

(12) $$d\phi_{i,L}=E\cdot \cos\left(\eta_{1}\right)\cdot dA$$

where the subindexes {*i,L*} stand for “input” and “Lambertian”, respectively, and the radiant intensity coming out of dA is

(13) $$dI_{e,L}=dI_{0,e,L}\cdot \cos\left(\eta_{2}\right)$$

where the subindex *e* stands for “exit”. The radiant intensity coming out of dA for $\eta_{2}=0$ is

(14) $$dI_{0,e,L}=\frac{R\cdot d\phi_{i,L}}{\int cos(\eta_{2})\cdot d\Omega }=\frac{R\cdot d\phi_{i,L}}{\pi }$$

where R is the reflectance of the plate. Finally, the radiant flux from dA reaching the detector is

(15) $$\begin{array}{cc} d\phi_{D} & =dI_{e,L}\cdot \Omega_{D}=dI_{0,e,L}\cdot \cos\left(\eta_{2}\right)\Omega_{D}=\frac{R\cdot d\phi_{i,L}}{\pi }\cdot \cos\left(\eta_{2}\right)\Omega_{D}=\\ \; & =\frac{R\cdot d\phi_{i,L}}{\pi }\cdot \cos\left(\eta_{2}\right)\frac{A\sigma }{r^{2}}=VWF\cdot \frac{R\cdot cos\left(\eta_{1}\right)\cdot cos\left(\eta_{2}\right)}{\pi }\cdot dA \end{array}$$

Therefore,

(16) $$L\left(x,y,z=h\right)=\frac{R\cdot cos\left(\eta_{1}\right)\cdot cos\left(\eta_{2}\right)}{\pi }$$

Dividing each VWF·L(x,y,z=h), obtained as the inverse Radon transform of the sinograms, by L(x,y,z=h), we obtain our experimental measurement of the volume weighting function, $VWF_{exp}\left(x,y,z\right)$. In conclusion, omitting for simplicity the dependence on (x,y,z),

(17) $$VWF=\frac{1}{L}R^{-1}\left[\frac{\Phi_{D}}{ds}\right]$$

#### 3.1.4. Experimental Determination of $W_{f}$

**Figure 6:**
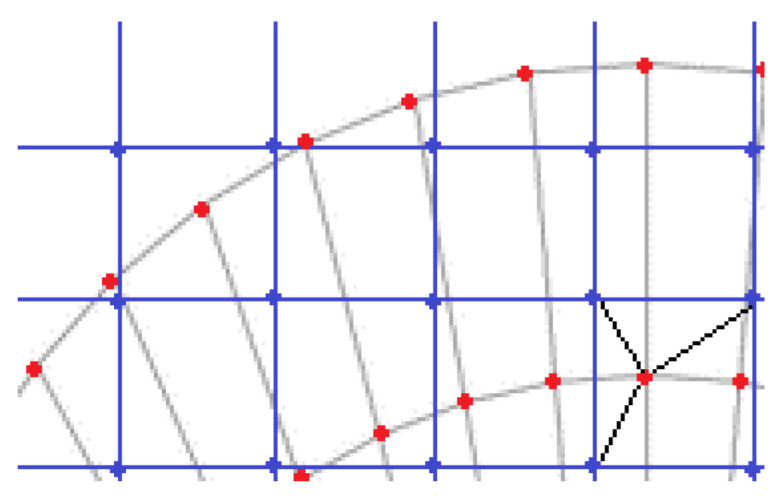
Interpolation Scheme: The blue mesh corresponds to a grid of points in the Cartesian system, where each point represents an experimental measurement on a section of the VWF. The red points are the values obtained through interpolation in the new coordinate system (q,p,θ).

To obtain $W_{f}$ as defined by Equation (7), it is necessary to express VWF(x,y,z) in a coordinate system ($q_{1}$, $q_{2}$, θ), where one of its coordinates is the scattering angle. This coordinate system has been defined in [19]. To numerically calculate the integral in (7), an interpolation of $VWF{\left(x,y,z\right)}_{exp}$ is performed on an (equispaced) mesh of the new coordinate system (Figure 6). Thus,

(18) $$W_{f}\left(\theta_{k}\right)=\sum_{i}\sum_{j}\Delta q_{1}\Delta q_{2}J\left(q_{1,i},q_{2,j},\theta_{k}\right)VWF\left(q_{1,i},q_{2,j},\theta_{k}\right)$$

In this expression, $\Delta q_{1}$ and $\Delta q_{2}$ is the mesh spacing at the coordinates $q_{1}$ and $q_{2}$, and $VWF(q_{1,i},q_{2,j},\theta_{k})$ are the experimental VWF interpolated at point $\left(q_{1,i},q_{2,j},\theta_{k}\right)$. Although points are equally spaced in the new coordinate system, in the Cartesian coordinate system they are not, which is the reason why the Jacobian determinant evaluated at the point $J(q_{1,i},q_{2,j},\theta_{k})$ must be included in the equation.

### 3.2. Spectral Dependence

In the previous subsection, the spectral dependence was omitted for simplicity, i.e, the problem was treated as if the source were monochromatic. In the real sensor, the source has a spectrum $E_{\lambda }$, and the detector has a spectral responsivity g(λ). Since the detector integrates in λ, the left-hand side of Equation (18) is multiplied by $\int_{\lambda }d\lambda \cdot E_{\lambda }\left(\lambda \right)g\left(\lambda \right)$, and the equation for $W_{f}$ needs to be divided by this factor:

(19) $$W_{f}\left(\theta_{k}\right)=\frac{\sum_{i}\sum_{j}\Delta q_{1}\Delta q_{2}J\left(q_{1,i},q_{2,j},\theta_{k}\right)VWF_{exp}\left(q_{1,i},q_{2,j},\theta_{k}\right)}{\int_{\lambda }d\lambda \cdot E_{\lambda }\left(\lambda \right)g\left(\lambda \right)}$$

## 4. Results

**Figure 7:**
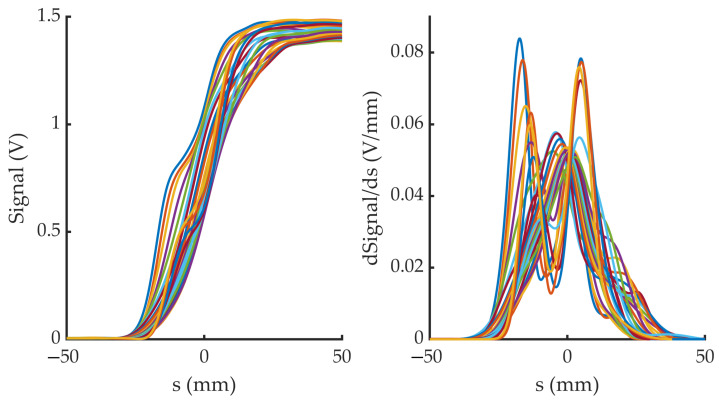
(**Left**): The 23 *s*-profiles (one for each α value) of signal measured for a fixed *h* distance (in this case h=11 mm. (**Right**): Derivatives of the previous profiles, after noise filtering.

The procedure described provides, for each distance *h* and each α value, a signal profile along the *s*-axis (see Figure 7, left). The next step is to obtain their s-derivatives, so that they can be interpreted as the line integral of a VWF section. Since the Radon transform is sensitive to noise, it is necessary to previously filter the noise measured by the instrument (in our case coming mainly from the electrical network). Figure 7 (right) shows this result.

**Figure 8:**
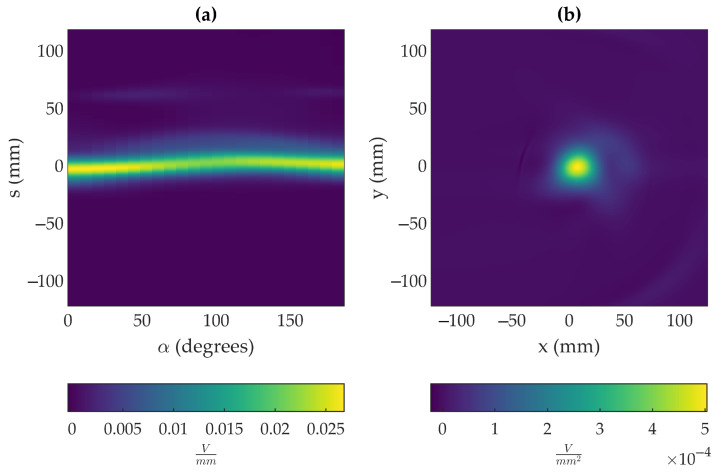
(**a**) Sinogram obtained for a distance *h* = 13 mm. (**b**) Inverse Radon transform applied to the sinogram.

All the signal derivatives for a fixed *h* value are joined together to form the sinogram (Figure 8a), whose inverse Radon transform shows the VWF with a superimposed Lambertian pattern. This Lambertian pattern is removed, as explained in Section 3.1.3, giving rise to a section of the VWF (Figure 8b).

**Figure 9:**
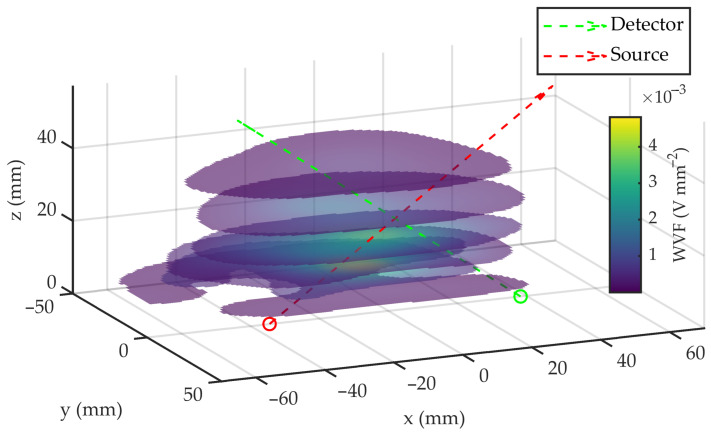
Schematics of the VWF sections obtained at different heights *h*.

To map the VWF, a total of 11 sections were obtained, covering the following *z* positions: [10, 13, 16, 20, 30, 40, …, 100] mm. Figure 9 shows the mapping of the first 6 sections.

**Figure 10:**
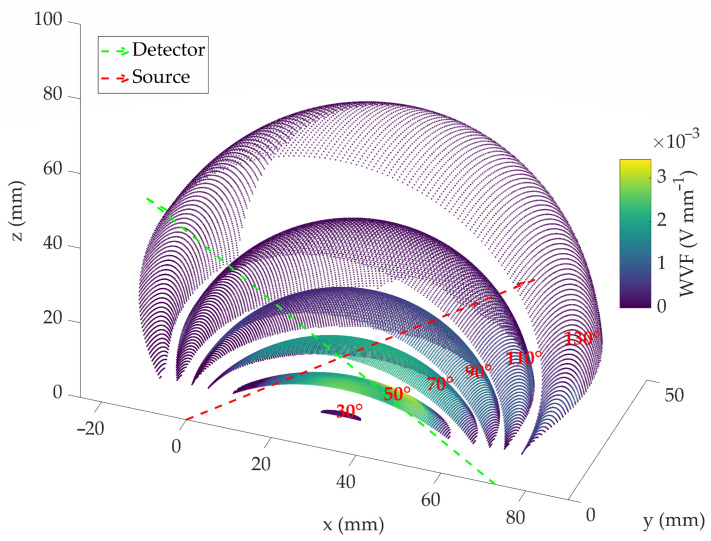
VWF interpolated for different scattering angle isosurfaces (represented in a Cartesian system).

Once the sections are obtained, it is necessary to interpolate them in the coordinate system ($q_{1}$, $q_{2}$, θ) mentioned before. This process is shown in Figure 10. In this figure, some of the interpolated isosurfaces can be observed (the rest are omitted for clarity reasons). Thus, the integral of VWF in one of these surfaces, or in other words, the sum of the values of its points (Equation (18)), gives rise to a specific value of $W_{f}$.

**Figure 11:**
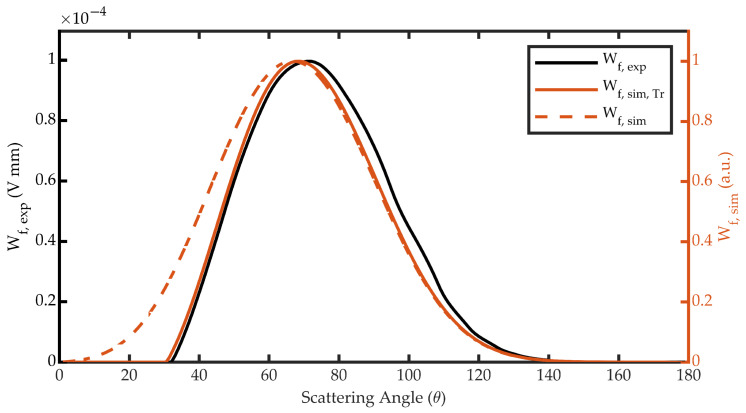
Angular weighting function of the forward configuration of the Dust Sensor.

Repeating this sum for all the isosurfaces results in $W_{f}\left(\theta_{k}\right)$ for the entire scattering angle interval (Figure 11). Although the figure shows that the intensity of VWF is higher in the 50-degree isosurface, the highest values of $W_{f}$ are found near 80 degrees. This is because, although more intense, this isosurface is smaller.

The following subsection includes an example of the application of the method to solve the direct problem (in the case of spherical particles).

### Particle Density Gain

In this example, a response factor of the instrument (*M*) to particles of a given radius R is calculated. The *M* value allows establishing a direct relationship between the signal that the instrument would provide and the density of particles in the interaction volume. The versatility of the method makes it possible to quickly calculate the sensitivity of the DS to particle size by simply calculating the M factor for different particle radii. The density of suspended dust is essential in various applications, including air quality monitoring, workplace safety and environmental studies. Since the volume scattering function β depends on the particle density ρ, our method can be applied to establish a relationship between the instrument signal and ρ. Since the functions of the instrument $W_{f}$ and g(λ) are already known, it is only necessary to express β as a function of ρ.

(20) $$\beta \left(\theta ,\lambda \right)=\rho \cdot \frac{\langle S_{11}\rangle \left(\theta ,\lambda \right)}{k^{2}}$$

where $S_{11}$ is the first element of the scattering matrix, which specifies the angular distribution of scattered light when the incident light is unpolarized, and k is the wavenumber. In this equation, $\langle S_{11}\rangle$ represent the statistical average over all the particle states of $S_{11}$.

This would allow us to calculate the particle density gain, M, for a given dust distribution.

(21) $$\langle Signal\rangle =\rho \cdot \int d\lambda \int d\theta \;W_{f}\left(\theta \right)\cdot g\left(\lambda \right)\cdot E_{\lambda }\left(\lambda \right)\cdot \frac{\langle S_{11}\rangle \left(\theta ,\lambda \right)}{k^{2}}=M\cdot \rho$$

Since *M* is to be calculated for different monodisperse distributions of spherical particles, statistical averaging is not required. In addition, the spherical symmetry allows to calculate the parameter $S_{11}$ from the Mie theory as a function of the radius of the particles *R* of each distribution and of the complex refractive index *m*; therefore, $\langle S_{11}\rangle \left(\theta ,\lambda \right)=S_{11}\left(R,m,\theta ,\lambda \right)$, and *M* can be expressed as

(22) $$M_{Mie}\left(R_{i}\right)=\int d\lambda \int d\theta \;W_{f}\left(\theta \right)\cdot g\left(\lambda \right)\cdot E_{\lambda }\left(\lambda \right)\cdot \frac{S_{11,Mie}\left(m,R_{i},\theta ,\lambda \right)}{k^{2}}$$

For these calculations, we used the complex refractive index for Martian dust proposed by [23] in the region between 1 and 5 microns. Specifically, the real part of the refractive index is 1.5, and the imaginary part can be neglected, since it is less than 1% in this region.

Table 1 shows the different values of gain in particle density *M* calculated for the Dust Sensor and spherical particles of different radii.

From the simulations carried out, a calibration table for spherical particles could be obtained. Then, knowing the radius of the particles, the density could be obtained.

It is important to note that the relationship between the instrument signal and particle density, ρ, through the volume scattering function β, can be established for various types of particles, regardless of their shape or size. The spherical case, such as raindrops, is just one example where this relationship can be expressed mathematically. In practice, however, the shape and size of the particles can significantly affect the scattering properties, making it necessary to consider more complex particle models. Nonetheless, our method provides a framework for determining the relationship between the instrument signal and particle density, allowing for the accurate measurement and analysis of suspended dust in various applications.

## 5. Discussion and Conclusions

In the previous section, the $W_{f}$ function for the forward configuration of the PbS sensor of the Dust Sensor was obtained experimentally. The results are in good agreement with the values obtained in a previous article [19], where it was found that the scattering angles measured in this configuration range roughly from 30 to 130 degrees.

However, the method proposed in this work has several advantages over the previous one:

- In [19], $W_{f}$ was calculated from the measurement of the irradiance pattern of the source and the angular sensitivity of the detector. Both were measured separately and then the detector and the IR source were assembled in the instrument. However, the assembly introduced modifications to the interaction volume geometry that the calculation did not take into account and had to be introduced a posteriori by applying a mask on the obtained $W_{f}$ function. In the present work, the angular function of the instrument, $W_{f}$, is measured experimentally, thus taking into account by design any effect of the final assembly reflected in the $W_{f}$.
- Moreover, as the $W_{f}$ is measured experimentally, it inherently includes a calibration factor that takes into account the effect of the optics and electronics of the system, and it avoids the use of specific calibration procedures.
- The novelty of the use of the tomographic techniques implemented in our method prevents assumptions or idealizations about the dimensions of the volume of interaction made. Being an experimental procedure, it realistically and quickly solves the direct model with a lower time investment than the time required by other simulation models, such as models based on Monte Carlo methods.

In addition:

- Although the aim here has been to determine the $W_{f}$ function, the method worked out implies the experimental determination of the volume of interaction and therefore is applicable of measuring the volume of interaction of other instruments.
- Another factor to consider is that the volume scattering function represents a single-scattering property. That is, the higher the particle density of the medium, the less reliable the use of $W_{f}$ will be. This assumption implies that the particles only scatter the light coming from the emitter (ignoring the scattered light from other particles). Thus, the lower the particle density of the medium, the more reliable the $W_{f}$ computed by this method [24].
- In Section 4, an application to relate the signal to a density of a spherical particles is shown. With the method proposed in this article, the instrument response factor (particle density gain) is calculated for spherical particles of different radii, which makes it possible to determine the sensitivity of the instrument to the radius of the particles. Although monodisperse distributions are not very realistic, this method will allow to simulate the signal in an equivalent way due to distributions of particles with variable radii. With this method, it will be possible to determine the $W_{f}$ function of the backward detector and calculate the signal that both detectors would receive for different distributions with varying densities and particle sizes. This extensive database will serve to solve the inverse problem and to relate the parameters that characterize the distributions (radius and effective variance) with the signals in the detectors in backward and forward configuration.

To conclude, the proposed method stands as an agile tool to solve the direct problem, i.e., to know the signal provided by the instrument given the characteristics of the particle distribution. Solving the direct problem is a key requirement to be able to design strategies and algorithms to solve the inverse problem, to carry out sensitivity studies and even to optimize the design of the instrument.

## Figures and Tables

**Table 1:** Particle density gain for different particle radii.

R [μm]	M [V/(part/cm${}^{3}$)]
0.5	$0.31\cdot 10^{-6}$
1	$3.0\cdot 10^{-6}$
2	$12\cdot 10^{-6}$
5	$42\cdot 10^{-6}$
10	$150\cdot 10^{-6}$
20	$520\cdot 10^{-6}$

**Figure A1:**
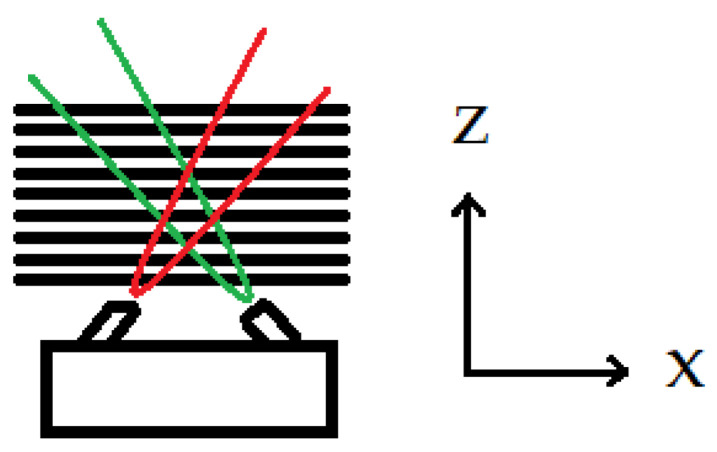
Mapping scheme of $WVF_{sim}$. The black lines indicate the Z positions where the function is evaluated.

**Figure A2:**
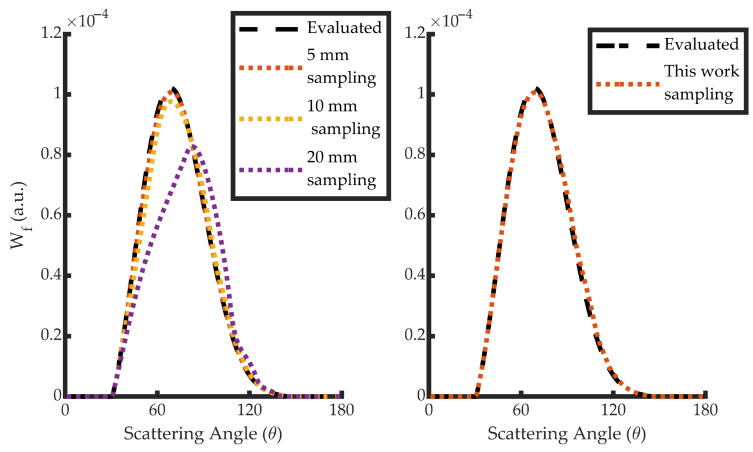
$W_{f}$ obtained by integrating $WVF_{sim}$. $WVF_{sim}$ was obtained by evaluating it in the coordinate system $\left(q_{1},q_{2},\theta \right)$, or by evaluating it in the Cartesian coordinate system with different samplings and interpolating it into the coordinate system $\left(q_{1},q_{2},\theta \right)$. (**Left**) Comparison of $W_{f}$ evaluated in $\left(q_{1},q_{2},\theta \right)$ with three $W_{f}$ mapped with equispaced z positions of 5, 10, and 20 mm. (**Right**) Comparison of $W_{f}$ evaluated in $\left(q_{1},q_{2},\theta \right)$ with $W_{f}$ mapped using the same scheme as in this work.

**Figure A3:**
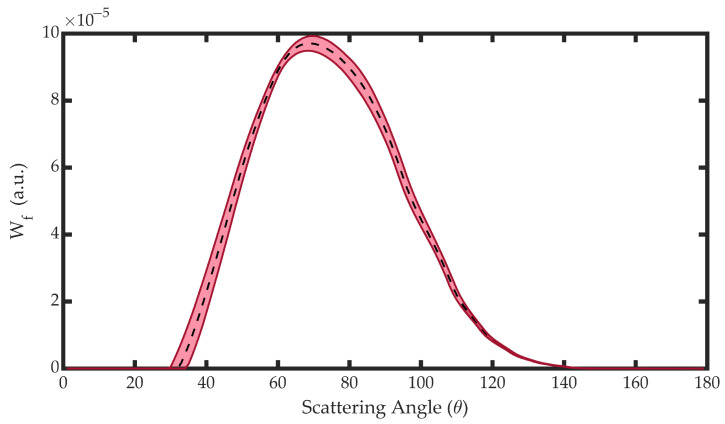
Confidence interval of 90%.

**Figure A4:**
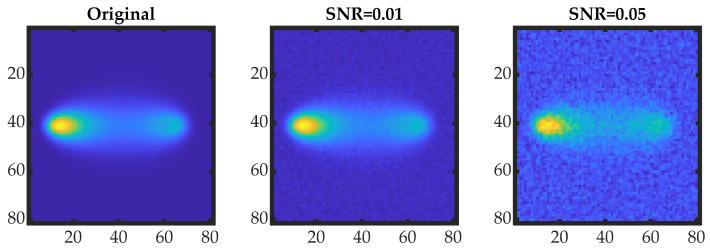
Reconstructed images from a sinogram without noise and with Gaussian noise with an SNR of 0.01 and 0.05.

**Figure A5:**
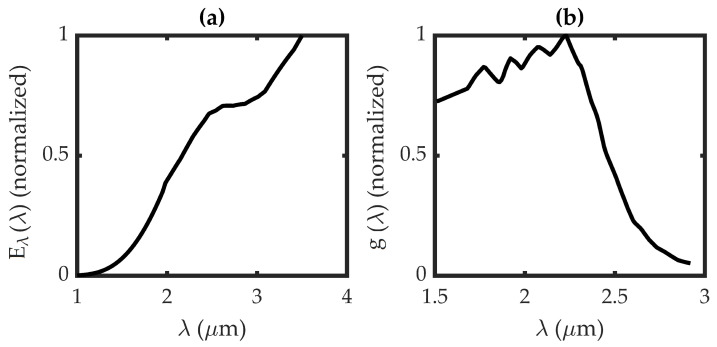
(**a**) E(λ). (**b**) g(λ).

**Figure A6:**
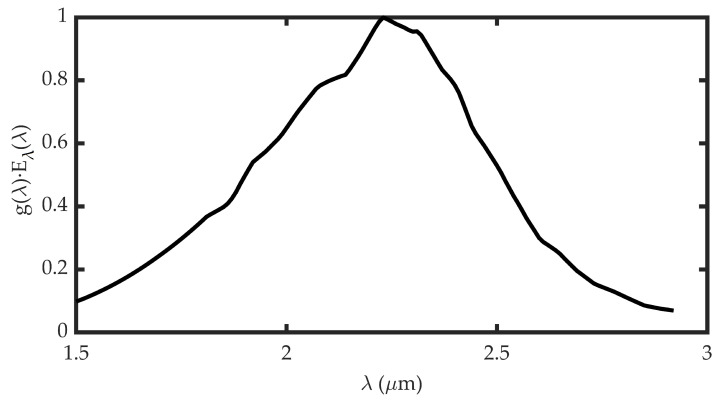
Product of g(λ) and $E_{\lambda }\left(\lambda \right)$.

## Data Availability

Not applicable.

## Appendix A. Error Estimation

In this appendix, three simulations are presented to estimate the error.

The first simulation focuses on the error associated with the mapping used to sample the interaction volume, specifically the target positions along the *z*-axis. The error associated with this discretization propagates during the interpolation and integration process of the VWF sections obtained in the new coordinate system. This study helps us estimate the optimal number of sections to be obtained and their positions, in other words, the optimal mapping.

To perform this simulation, a VWF (referred to as $WVF_{sim}$) was simulated using an emission pattern of the source $E_{0}\left(x,y,z\right)$ and a detector angular sensitivity $\sigma (\gamma_{D}\left(x,y,z\right))$ that were experimentally determined and can be found in the appendix in [19].

This VWF was evaluated in two different ways:

- In the coordinate system $\left(q_{1},q_{2},\theta \right)$, where its integral provides us with a ground truth $W_{f}$.
- In the Cartesian coordinate system (Figure A1). This allows us to emulate the error introduced during the interpolation described in Section 3.1.4.

Figure A2 compares the $W_{f}$ evaluated in $\left(q_{1},q_{2},\theta \right)$ with the interpolated $WVF_{sim}$ using different mappings. As shown in Figure A2 (left), a mapping with a 5 mm interval perfectly reproduces the $WVF_{sim}$. In fact, if we compare the mapping used in this work with the $WVF_{sim}$, Figure A2 (left), we can observe that the error induced by the mapping is negligible.

The second simulation aims to estimate the tolerance of the mapping, or in other words, determine the error in $W_{f}$ associated with the uncertainty in the positioning of the different sections.

For this simulation, $WVF_{sim}$ was evaluated using the same mapping as in this work (X=Y=[−100,−99,⋯,99,100] mm, Z=[10,13,16,20,30,40,…,100] mm).

To obtain $W_{f}$, it is necessary to interpolate $WVF_{sim}$ in the $\left(q_{1},q_{2},\theta \right)$ coordinate system. To estimate the uncertainty in $W_{f}$ caused by the uncertainty in the placement of the Lambertian target, we added random uncertainty, Δz, to each Z position before interpolating $WVF_{sim}$. As a result, the integrated interpolated VWF will exhibit a small error. This process can be repeated a large number of times to estimate the uncertainty of $W_{f}$.

Figure A3 shows the median and 90% confidence interval after 1000 simulations. Δz was randomly generated for each simulation and each position in the *Z* vector following a normal distribution with a standard deviation of 0.5 mm, which is the estimated uncertainty in *Z* according to the experimental setup described in Section 3.1.1.

Finally, due to the sensitivity of the inverse Radon transform to noise, we want to emphasize the importance of obtaining noise-free sinograms. To achieve this, a sinogram was simulated and Gaussian noise was added to it, equivalent to an SNR of 0.01 and 0.05. The results are shown in Figure A4, where it can be observed that an SNR of 0.05 significantly degrades the obtained sinogram.

## Appendix B. Spectral Characteristics

In this appendix, we present the spectral characteristics of the source and the detector.

The emitter is the JSIR-350, a thermal emitter with behavior similar to that of a gray body [25]. Since it implements lock-in amplification, it is necessary to consider the processing described in [22]. $E_{\lambda }$ is shown in Figure A5a.

The detector is a PBS on which an interferential filter from 1 to 3 um was deposited. g(λ) is calculated as the product of the specific detectivity of the PBS at the experimental temperature and the transmittance of the filter, shown in Figure A5b.

To fully determine the direct model, it is necessary to know the spectral factor included in Equation (19). The product between the spectral functions $E_{\lambda }$ and g(λ) is shown in Figure A6.
